# Association Study of Alcohol Dehydrogenase and Aldehyde Dehydrogenase Polymorphism With Alzheimer Disease in the Taiwanese Population

**DOI:** 10.3389/fnins.2021.625885

**Published:** 2021-01-22

**Authors:** Yah-Yuan Wu, Yun-Shien Lee, Yu-Li Liu, Wen-Chuin Hsu, Wei-Min Ho, Yu-Hua Huang, Shih-Jen Tsai, Po-Hsiu Kuo, Yi-Chun Chen

**Affiliations:** ^1^Department of Neurology, Chang Gung Memorial Hospital, Linkou Medical Center and College of Medicine, Chang Gung University, Taoyuan, Taiwan; ^2^Department of Biotechnology, Ming Chuan University, Taoyuan, Taiwan; ^3^Genomic Medicine Research Core Laboratory, Chang Gung Memorial Hospital, Taoyuan, Taiwan; ^4^Center for Neuropsychiatric Research, National Health Research Institutes, Zhunan, Taiwan; ^5^Department of Psychiatry, Taipei Veterans General Hospital, Taipei, Taiwan; ^6^Division of Psychiatry, National Yang-Ming University, Taipei, Taiwan; ^7^Department of Public Health, Institute of Epidemiology and Preventive Medicine, National Taiwan University, Taipei, Taiwan; ^8^Department of Psychiatry, National Taiwan University Hospital, Taipei, Taiwan

**Keywords:** alcohol dehydrogenase, aldehyde dehydrogenase, Alzheimer’s disease, ADH1C level, *ADH1C* rs2241894

## Abstract

Alcohol dehydrogenase (ADH) and aldehyde dehydrogenase (ALDH) are two major alcohol-metabolizing enzymes. Moderate alcohol intake is a protective modified factor in Alzheimer’s disease (AD) while heavy alcohol intake and abstinence increased dementia risk. The associations between Alzheimer’s disease and alcohol-metabolizing genes are uncertain. This study examined the association of AD with seven *ADH/ALDH* single-nucleotide polymorphisms (SNPs), *ADH1C* rs2241894, *ADH1B* rs1229984, *ALDH1B1* rs2073478, *ALDH2* rs886205, rs4767944, rs4648328, and rs671. We enrolled 157 AD and 168 age- and sex-matched control subjects in pilot study to examine the association of AD with *ADH/ALDH* SNPs. Reconstructed *ALDH2* haplotypes were performed. We measured plasma level of ADH1C and checked the interaction effect of AD–rs2241894 genotype on plasma ADH1C level. In extension study, we further examined 339 AD and 2,504 healthy control from the Taiwan Biobank. In pilot study, we observed that *ADH1C* rs2241894 TT genotype was negatively associated with AD in a recessive genetic model (OR = 0.25, 95% CI 0.09–0.75, *p* < 0.0001) in women. A strong linkage disequilibrium was observed among the four examined SNPs of *ALDH2*. No haplotype was related to AD. The plasma ADH1C level in AD was higher than that in control. After adjusted by age, sex, hypertension, diabetes mellitus, and alcohol, we found a significant interaction effect of AD–rs2241894 genotype on plasma ADH1C level (*p* = 0.04). This interaction effect was attributable to the association between AD and plasma ADH1C level (β estimate = 366, 95% CI 92.7∼639.4, *p* = 0.009). The genetic distribution of *ADH1C* rs2241894 showed strong ethnic heterogeneity, in which the T allele was the minor allele accounting for 28.5% in our study and 23.6% in East Asians, while it was a major allele in Americans, Europeans, and the global populations. No association was discovered between AD and the five SNPs: rs2241894, rs1229984, rs2073478, rs886205, and rs671 in the extension study. In summary, this study revealed a suggestive association between ADH1C rs2241894 and female AD in the pilot study, but failed to confirm this finding in a population database. Further age-matched and large sample size case-control studies are needed before rs2241894 can be interpreted as a protective genetic factor of AD.

## Introduction

Alzheimer’s disease (AD) is the leading cause of dementia, especially in the elderly. The prevalence is 40.2 per 1,000 persons in participants older than 60 years in the community (Fiest et al., 2016). The incidence of the disease doubles every 5 years after 65 years of age (Querfurth and LaFerla, 2010; Hebert et al., 2013). Patients suffer from cognitive decline and eventually progress to loss of daily function or death. Amyloid and tau deposition causes oxidative stress and worsens mitochondrial and synaptic dysfunction (Querfurth and LaFerla, 2010). Non-modifiable risk factors of AD include aging, female sex, and genetic risks, such as carrying the apolipoprotein E (APOE) e4 allele (Corder et al., 1993), whereas risk factors, such as diabetes mellitus (DM), hypertension (HTN), obesity, smoking, excessive alcohol consumption over 168 g weekly, and educational attainment, are potentially modifiable (Lourida et al., 2019).

Alcohol abuse is a major factor in brain damage (Ridley et al., 2013). According to the Centers for Disease Control and Prevention (CDC) of the United States, heavier drinkers were defined as those consuming more than 28 g of pure alcohol per day in the case of men and 14 g in the case of women (Schoenborn et al., 2013). Fourteen grams of pure alcohol corresponds to a 12-US-fluid-ounce (350 mL) glass of 4.1% beer or a 5-US-fluid-ounce (150 mL) glass of 12% alcohol-by-volume wine. A 23-year follow-up prospective cohort study suggested that alcohol consumption is a risk factor for dementia in both sexes. The effect of alcohol consumption and dementia is considered to be J-shaped, where excessive alcohol intake (>112 g/week) or abstinence increased dementia risk, compared with consuming 9–112 g/week (Sabia et al., 2018). The J-shaped effect was also observed between alcohol and AD. In systemic reviews, drinking patterns are associated with AD and mild cognitive impairment, where abstinence and heavy drinking were associated with an increased risk of AD onset compared with moderate drinking (Rehm et al., 2019).

Alcohol is primarily metabolized by alcohol dehydrogenase (ADH) and aldehyde dehydrogenase (ALDH). The metabolism of alcohol produces acetaldehyde, acetate, and reactive oxygen species. Both high ADH activity and low ALDH activity cause an excess of acetaldehyde and result in oxidative stress (Ohta et al., 2004). ADH families (EC 1.1.1.1) are a group of dehydrogenase enzymes that facilitate the interconversion between alcohols and aldehydes or ketones with the reduction of NAD + to NADH during the biosynthesis of various metabolites. ALDH families (EC 1.2.1.3) are a group of enzymes that catalyze the oxidation of aldehydes (Cederbaum, 2012). ADH and ALDH variants have been shown to influence alcohol dependence in previous studies (Sun et al., 2019). Lacunar infarction (Suzuki et al., 2004) and neuropsychiatric disease were observed to be associated with the ADH genotype, whereas Parkinson’s disease was not (Suzuki et al., 2004; Garcia-Martin et al., 2019; Kim et al., 2020). On the other hand, *ALDH2* polymorphism is related to Parkinson’s disease and intracranial hemorrhage (Chen et al., 2019; Huang et al., 2020). The prevalence of the *ADH1B*, *ADH1C*, and *ALDH2* genotypes is higher in Asians (Eng et al., 2007). In the literature review, approximately 30% of people in Asia and 47% of people in Taiwan were found to carry genetic variants of the *ALDH2* A allele by rs671 with reduced enzymatic activity (Chiang et al., 2016). *ALDH1B1*, which shares significant sequence homology with *ALDH2*, is related to drinking habits in Caucasians (Husemoen et al., 2008). The association between *ALDH2* genetic polymorphisms and AD was inconclusive. Some reports suggested that *ALDH2^∗^2* (rs671 variation) is a risk factor for AD in Japanese (Kamino et al., 2000), whereas others reported no association in Japanese and older Korean populations (Kim et al., 2004; Komatsu et al., 2014). *ALDH2* rs886205 affects the methylation of the *ALDH2* premotor region (Pathak et al., 2017). Meta-analysis showed a borderline influence of *ALDH2^∗^2* on AD in a recessive genetic fashion (Hao et al., 2011; Chen et al., 2019), but it was not identified as a true susceptibility AD gene among the 695 gene candidates in the AlzGene database. To date, there have been no studies addressing *ALDH1B1* polymorphisms and AD risks. In ADH1C, the rate of alcohol elimination was proved to be associated with the SNPs across ADH1C and ADH1B (Birley et al., 2009). From GWAS (genome-wide association) of alcohol dependence in African- American, *ADH1C* rs2241894 (p.Thr151 =) is a risk loci mapped to alcohol-metabolizing enzyme genes (Gelernter et al., 2014). No report of the association between AD and ADH1C SNPs so far.

We selected *ADH1C* rs2241894 (*ADH1C*, chr4: 99344976, Synonymous Variant, c.453 A > T,C, p.Thr151 =), *ADH1B* rs1229984 (chr4:99318162, Missense Variant, c.143A > G, p.His48Arg), *ALDH1B1* rs2073478 (chr9:38396068, c.320G > A, p.Arg107His), *ALDH2* rs886205 (G > A, promoter, 5’-untranslated region), *ALDH2* rs4767944 (C > G,T, Intron Variant), *ALDH2* rs4648328 (C > T, intron variant, intron 3), and *ALHD2* rs671 (G > A, missense variant Glu504Lys, exon 12) based on previous evidence of their association with alcohol dependence (Edenberg and Foroud, 2013).

Only a few studies have addressed the associations between AD and alcohol-metabolizing enzymes. Given that the ADH/ALDH pathway is involved in numerous risks of AD, including oxidative stress, HTN, and alcohol consumption, this study first utilized single-center case-control data for evaluation, and then used Taiwan population genomic data for replication analyses to examine whether these genes are AD-susceptible genes. This study was extended to ALDH-related pathways, which is a novel route to examine the association between AD and the ADH gene.

## Materials and Methods

### Patient and Control Subject Recruitment

This study was designed as a two-step process. First, we enrolled 157 AD patients and 168 age- and sex-matched control subjects in a pilot study. AD diagnosis was made according to the 2011 diagnostic criteria of the National Institute on Aging-Alzheimer’s Association workgroups (NIAAA) (McKhann et al., 2011). The control group consisted of sex- and age-matched subjects who visited Chang Gung Memory Hospital (CGMH) for a health exam or treatment for diseases other than neurodegenerative diseases or cerebrovascular diseases.

Second, the number of AD patients was expanded to 339. A total of 2504 healthy participants selected from the Taiwan Biobank were included in the extension study. The Taiwan Biobank is a prospective population-based study that enrolled healthy seniors with extensive baseline phenotypic measurements, genomic data, and stored biological samples. The criteria for selecting the control groups from the Taiwan Biobank were the age range 50–70 years, no history of stroke or dementia, and self-reporting as being of Taiwanese Han Chinese ancestry (Chen et al., 2016). Details on the Taiwan Biobank can be found on its official website^1^.

### Selection of SNPs, Genotyping, and Haplotype Construction for Cases and Control

Based on a previously reported association with alcohol dependence (Edenberg and Foroud, 2013), the pilot study analyzed seven SNPs, namely, *ADH1C* rs2241894, *ADH1B* rs1229984, *ALDH1B1* rs2073478, *ALDH2* rs886205, *ALDH2* rs4767944, *ALDH2* rs4648328, and *ALHD2* rs671. Only 5 SNPS were evaluated in extension study because custom Taiwan Biobank chips (Affymetrix, Santa Clara, CA, United States) only contains only 5 ones, namely *ADH1C* rs2241894, *ADH1B* rs1229984, *ALDH1B1* rs2073478, *ALDH2* rs886205. Among these SNPs, *ADH1C* rs2241894 is believed to affect alcohol metabolism. *ADH1B* rs1229984 is a well-studied genetic variant associated with alcohol dependence in Asians. Genomic DNA was extracted from peripheral leukocytes using the Stratagene DNA extraction kit (La Jolla, CA, United States). SNP polymorphisms were genotyped using TaqMan^®^ Assays in the ABI Prism 7900HT Sequence Detection System (catalog #4317596, Applied Biosystems, Foster City, CA, United States) (Schleinitz et al., 2011). Plasma ADH1C level was determined using human ADH1C ELISA kit (catalog #MBS2889930, MyBioSource, San Diego, CA, United States) and monitored spectrophotometrically at 450 nm on a multifunctional microplate reader (Tecan infinite 200) by following the manufacturer’s instructions. Levels of ADH1C were determined from a standard curve. Patterns of linkage disequilibrium (LD) were evaluated using Haploview v4, and haplotypes were reconstructed using PHASE 2.0 (Barrett et al., 2005) based on the LD results. Haplotypes with a frequency <1% were excluded from the association analysis. In participants from the Taiwan Biobank, SNP genotypes were obtained from the data derived from the custom Taiwan Biobank chips and run on the Axiom Genome-Wide Array Plate System (Affymetrix, Santa Clara, CA, United States).

### Statistical Analysis and Power Estimation

Pearson’s χ2-test or *t*-test was used to compare the demographic data and the distributions of genotypes of AD and control. Two-tailed *p*-values were derived from the χ2-test or Fisher’s exact test. Association analyses were performed stratified by sex. Hardy–Weinberg equilibrium was performed via χ2-test for all SNPs at a significance level of 0.05. Multivariable logistic regression was used to analyze the phenotype-genotype associations of AD with ADH and ALDH alleles under dominant, recessive, and additive genetic models. The covariables included age, years of education, HTN, DM, and alcohol use. Since considering Bonferroni correction, the significance level was set to 0.007 in pilot study and 0.01 in extension study. The permutation testing was performed when the *p*-value was under Bonferroni correction in pilot study. Analysis of interaction effect (Chen et al., 2009) was performed to evaluate how carrying APOE ε4 influence the *ADH1C* rs2241894 to AD susceptibility, because APOE ε4 and ADH1C shared the common pathway of oxidative stress. All the data analyses were performed using SAS software version 9.1.3 (SAS Institute, Cary, NC, United States). Association of the interaction effect between AD and rs2241894 genotypes on the plasma ADH1C level was tested by the general linear models (GLM) with adjustment for age, sex, DM, HTN, and alcohol. We also perform analysis of interaction effect of AD-rs2241894 genotype in ADH1C level.

We evaluated the ability to detect an association between an SNP and AD via a power calculation implemented in QUANTO version 1.0 (Gauderman, 2002). When Minor allele frequency (MAF) > 0.2 under a recessive genetic model at a significance level of 5%, we observed that the power to identify an association was greater than 0.8 when the per-allele genetic effect was greater than 3.5 and 2.0 in the pilot case-control study and in the extension study, respectively.

## Results

### Demography of the Pilot Case-Control Study

A total of 157 AD patients and 168 controls were included in the pilot study (Table 1). The years of education were higher in the female AD patients than in the controls. The age and sex between the AD patients and the controls were matched in this dataset. The proportion of APOE ε4 carriers was higher in the AD patients than in the controls. The proportion of DM was higher in the female patients with AD than in the controls. There were no differences in age, HTN frequency, and the proportion of alcohol use. As the proportion of alcohol use was remarkably different between sexes, the analyses were stratified by sex.

**TABLE 1:** Background demographic distribution and frequency of the genotype in the pilot study.

	Males (*n* = 147)			Females (*n* = 178)		
	AD	Controls	*p*-Value	AD	Controls	*p*-Value
	(*n* = 73)	(*n* = 74)		(*n* = 84)	(*n* = 94)	
Age (years)	69.4 ± 9.0	67.1 ± 5.3	0.06	65.4 ± 5.9	67.0 ± 6.3	0.08
Education (years)	8.4 ± 4.1	9.3 ± 4.7	0.31	7.4 ± 4.5	5.6 ± 4.7	0.01
Hypertension (%)	55.40%	52.51%	0.69	45.2%	44.7%	0.94
Diabetes mellitus (%)	20.30%	21.10%	0.9	36.9%	20.2%	0.01
Alcohol use (%)	17.60%	16.90%	0.92	1.2%	1.1%	0.94
APOE ε4 carrier	*n* = 73 30.1%	*n* = 57 12.3%	0.02	*n* = 84 43.4%	*n* = 83 21.7%	0.01
*ADH1B*						
rs1229984 TT/TC/CC	49.4/39.5/11.1	56.2/41.1/2.7	0.14	60.7/35.7/3.6	53.2/42.6/4.3	0.6
*ADH1C*						
rs2241894 CC/CT/TT	43.8/42.5/13.7	52.1/38.4/9.6	0.55	54.8/44.0/1.2	46.8/43.6/9.6	0.05
*ALDH1B1*						
rs2073478 GG/GT/TT	47.9/36.6/15.5	52.8/31.9/15.3	0.82	41.0/51.8/7.2	48.4/36.6/15.1	0.07
*ALDH2*						
rs886205 GG/GA/AA	77.0/21.6/1.4	78.1/20.5/1.4	0.99	82.1/17.9/0.0	74.5/22.3/3.2	0.18
rs4767944 TT/TC/CC	48.6/41.9/9.5	41.1/49.3/9.6	0.63	51.8/41.0/7.2	47.8/38.0/14.1	0.34
rs4648328 CC/CT/TT	67.1/28.8/4.1	57.5/38.4/4.1	0.46	68.7/26.5/4.8	64.9/31.9/3.2	0.66
rs671 GG/GA/AA	46.6/35.6/17.8	46.6/42.5/11.0	0.44	47.6/41.7/10.7	41.5/50.0/8.5	0.53

**N*, number, AD, Alzheimer disease, ADH, alcohol dehydrogenase, ALDH, aldehyde dehydrogenase. Data are expressed as percentage or mean ± SE. Comparisons between AD cases and controls were analyzed using the χ2 test or *t*-test where appropriate.*

### Genotype Frequency and Association Analysis of the Pilot Case-Control Study

All seven SNPs were in Hardy–Weinberg equilibrium at a significance level of 0.05. The frequencies of each genotype in the AD and control subjects are listed in Table 1. The proportion of *ADH1C* rs2241894 TT genotype (minor allele T) was significantly lower in the female patients with AD than in the female controls. The association between the SNP genotype and AD is presented in Table 2. In the female group, *ADH1C* rs2241894 was significantly associated with AD in the recessive genetic model (OR = 0.25, 95% CI 0.09–0.75, *p* < 0.0001). APOE ε4 carriers had no interactive effect between AD and *ADH1C* rs2241894. This study did not find an association between AD and the other six SNPs in the female groups and any candidate SNPs in the male groups (Supplementary Table 1).

**TABLE 2:** Associations of the candidate SNPs with AD in the pilot study.

Gene	SNP	Position	Dominant *p*-values	Additive *p*-values, (OR, 95% CI)	Recessive *p*-values, (OR, 95% CI)
***ADH1B***: Missense Variantc.143A > G, p.His48Arg	rs1229984	chr4:99318162	M: 0.496, F: 0.04	M: 0.23, F: 0.07	M: 0.12, F: 0.99
***ADH1C***: Synonymous Variant, c.453 T > A,C, p.Thr151 =	rs2241894	chr4:99344976	M: 0.487, F: 0.256	M: 0.45, F: 0.04	M: 0.06, *F* < 0.0001 (0.25, 0.09–0.75)
***ALDH1B1***: Missense Variant, c.320G > A, p.Arg107His	rs2073478	chr9:38396068	M: 0.66, F: 0.25	M: 0.52, F: 0.83	M: 0.48, F: 0.15
***ALDH2***: 2KB Upstream Variant, A > G	rs886205	chr12:111766623	M: 0.788, F: 0.193	M: 0.77, F: 0.08	M: 0.84, F: NA
***ALDH2***: Intron Variant, C > G,T	rs4767944	chr12:111771537	M: 0.205, F: 0.661	M: 0.43, F: 0.39	M: 0.64, F: 0.24
***ALDH2***: Intron Variant, C > T	rs4648328	chr12:111784984	M: 0.338, F: 0.707	M: 0.45, F: 0.92	M: 0.88, F: 0.53
***ALDH2***: Missense Variant, c.1510G > A, p.Glu504Lys	rs671	chr12:111803962	M: 0.987, F: 0.388	M: 0.69, F: 0.55	M: 0.41, F: 0.91

*M, male, F, female, NA, not applicable, OR, odds ratio, 95% CI = 95% confidence interval. Logistic regression was performed, adjusting for age, education, HTN, DM, and alcohol use.*

**P*-value with Bonferroni correction for significance was 0.007. The permutation testing of 10,000 replicates was performed when the *p*-value was significant under Bonferroni correction.*

### Demography of the Extension Study

Among 339 AD patients and 2,504 control subjects in the extension cohort study, there were 123 AD and 1,271 controls in men (Table 3). The AD patients were older than the control subjects. Among men, the mean age was 71.0 (± 10.1) in the AD group and 64.1 (± 2.8) years in the control group (*p* < 0.05). Among women, the mean age was 72.6 (± 8.5) in the AD group and 64.0 (± 3.0) in the control group (*p* < 0.05). The years of education were higher in the AD group than in the control group (AD 8.6 ± 4.3, control 5.2 ± 1.2, *p* < 0.05). The proportions of HTN and DM were higher in the AD group than in the control group (HTN AD 50.5%, control 35.4%, *p* < 0.05, DM AD 37.5%, control 8.8%, *p* < 0.05). As the proportion of alcohol use was remarkably different between sexes, the analyses were stratified by sex.

**TABLE 3:** Background demographic distribution and frequency of the genotype in the extension study.

	Males (*n* = 1394)			Females (*n* = 1449)		
	AD	Controls	*p*-Value	AD	Controls	*p*-Value
	(*n* = 123)	(*n* = 1271)		(*n* = 216)	(*n* = 1233)	
Age (years)	71.0 ± 10.1	64.1 ± 2.8	2.3E-66	72.6 ± 8.5	64.0 ± 3.0	8.06E-134
Education (years)	8.6 ± 4.3	5.2 ± 1.2	1.1E-85	4.9 ± 4.6	4.6 ± 1.3	0.06
Hypertension (%)	48.8%	45.6%	0.50	50.5%	35.4%	2.40E-5
Diabetes mellitus (%)	22.0%	14.0%	0.17	37.5%	8.8%	5.80E-31
Alcohol use (%)	14.6%	11.7%	0.35	0.9%	1.1%	0.86
*ADH1B*						
rs1229984 TT/TC/CC	54.1/35.2/10.7	54.2/39.3/6.6	0.21	48.3/46.40/5.2	54.7/39.3/6.0	0.14
ADH1C						
rs2241894 CC/CT/TT	46.7/46.2/10.7	51.2/42.3/6.6	0.21	57.1/37.2/5.7	55.3/38.1/6.6	0.38
*ALDH1B1*						
rs2073478 GG/GT/TT	50.8/35.0/14.2	46.1/42.9/11.0	0.21	42.4/48.1/9.5	46.7/42.8/10.5	0.35
*ALDH2*						
rs886205 GG/GA/AA	77.3/20.3/2.4	77.9/20.8/1.3	0.62	76.5/22.5/0.9	77.5/21.5/1.0	0.90
rs671 GG/GA/AA	50.8/35.2/13.9	50.6/40.8/8.6	0.11	49.1/40.6/10.4	50.6/40.8/8.5	0.67

**n*, number; AD, Alzheimer disease; ADH, alcohol dehydrogenase; ALDH, aldehyde dehydrogenase. Data are expressed as percentage or mean ± SE. Comparisons between AD cases and controls were analyzed using the χ2 test or *t*-test where appropriate.*

### Genotype Frequency and Association Analysis of the Extension Study

All five SNPs were in the Hardy–Weinberg equilibrium at a significance level of 0.05. The frequencies of each genotype in the AD and control subjects are listed in Table 3. The association between the SNP genotype and AD is presented in Table 4. The *ADH1C* rs2241894 genotype had no association with AD after adjusting for age, years of education, proportion of alcohol use, and comorbidities in both sexes. No association was discovered between AD and the other SNPs (Supplementary Table 2).

**TABLE 4:** Associations of the candidate SNPs with AD in the extension study.

Gene	SNP	Position	Dominant *p*-values	Additive *p*-values	Recessive *p*-values, (OR, 95% CI)
***ADH1B***: Missense Variantc.143A > G, p.His48Arg	rs1229984	chr4:99318162	M: 0.46, F: 0.78	M: 0.41, F: 1.00	M: 0.58, F: 0.59
***ADH1C***: Synonymous Variant, c.453T > A,C p.Thr151 =	rs2241894	chr4:99344976	M: 0.24, F: 0.39	M: 0.09, F: 0.29	M: 0.08, F: 0.72
***ALDH1B1***: Missense Variant, c.320G > A, p.Arg107His	rs2073478	chr9:38396068	M: 0.12, F: 0.12	M: 0.34, F: 0.40	M: 0.72, F: 0.77
***ALDH2***: 2KB Upstream Variant, A > G	rs886205	chr12:111766623	M: 0.48 F: 0.51	M: 0.19, F: 0.46	M: NA, F: 0.60
***ALDH2***: Missense Variant, c.1510G > A, p.Glu504Lys	rs671	chr12:111803962	M: 0.57, F: 0.65	M: 0.39, F: 0.56	M: 0.29, F: 0.59

*M, male, F, female, NA, not applicable, OR, odds ratio, 95% CI = 95% confidence interval, Logistic regression was performed, adjusting for age, education, HTN, DM, and alcohol use. *p*-value with Bonferroni correction for significance was 0.01.*

### Haploview Analysis of *ALDH2* SNPs

**FIGURE 1:**
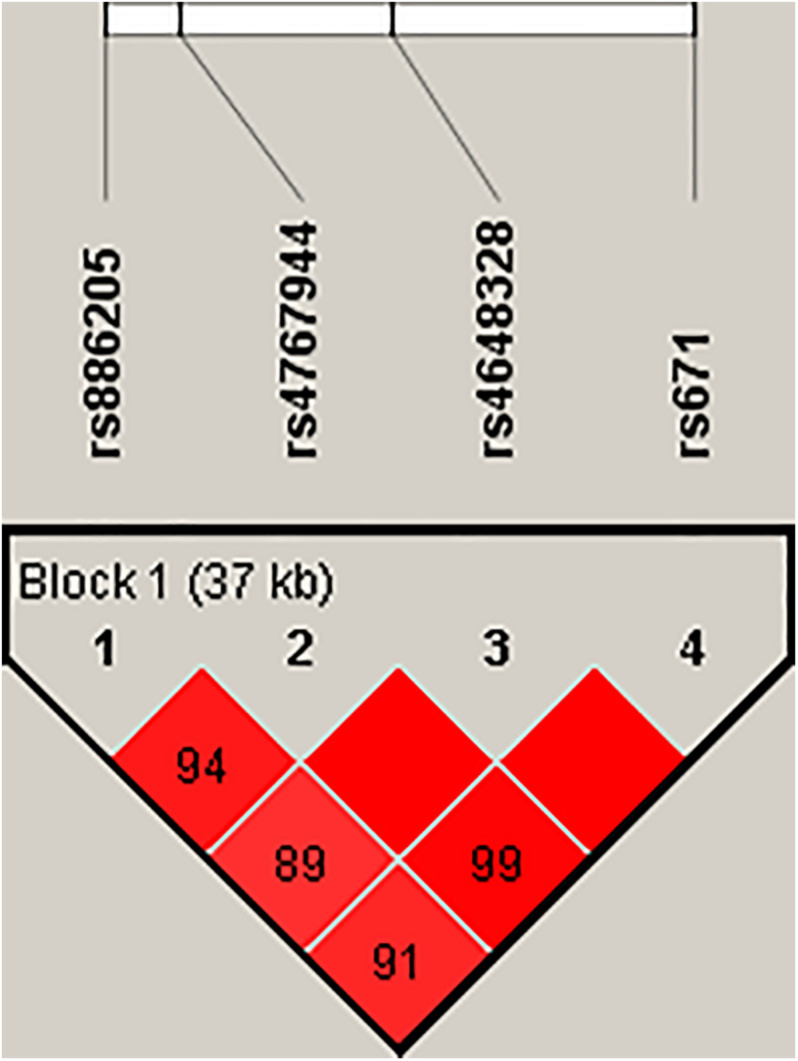
Haploview disequilibrium coefficients (D’) of the pairwise loci in the AD and control groups. Four SNPs in the genomic region of *ALDH2* loci were analyzed using Haploview version 4.2 software. Strong LD was observed among rs886205, rs4767944, rs4648328, and rs671. Four SNPs constituted one haplotype block that span 37 kb. A D’ value of “0” indicates the independence of the two examined loci, whereas a value of “1” demonstrates complete linkage. The strength of LD is depicted by the intensity of red color. It changes from white to red as D’ × 100 progresses from 1 to 100.

In the pilot case-control study, a haplotype block of *ALDH2* was further constructed by rs886205, rs4767944, rs4648328, and rs671 using Haploview (4.2), where there was one block with strong LD (Figure 1). In the haplotype analyses, there was no association between the haplotype and AD susceptibility.

### Plasma ADH1C Level

**FIGURE 2:**
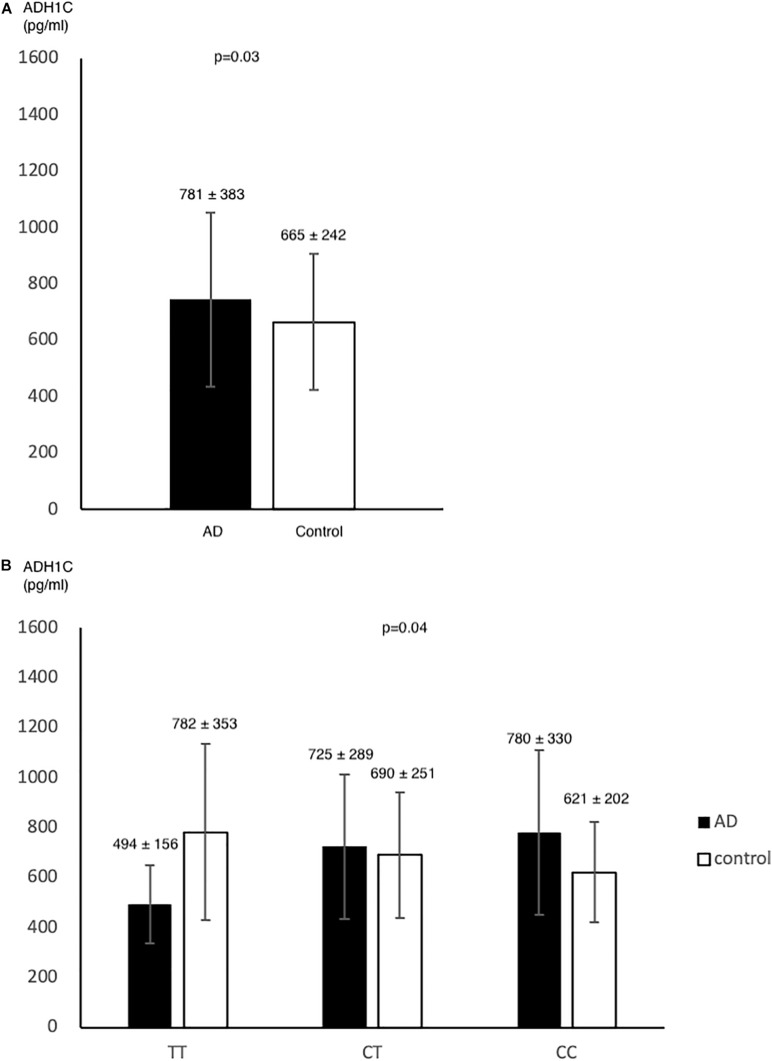
ADH1C level in AD and control. Plasma ADH1C level was higher in AD than in control **(A)**. **(B)** there was a marginal interaction effect between AD and rs2241894 genotype on plasma ADH1C level (*p* = 0.04), in which those carried minor allele T had lower ADH1C level in AD patients but higher ADH1C level in the controls.

In pilot case-control study, we examined the plasma level of ADH1C. AD had higher ADH1C level in comparison to control group (*n* = 78, *n* = 72, 781 ± 383, 665 ± 242, respectively) (*p* = 0.03) (Figure 2A). After adjusted by age, sex, HTN, DM, and alcohol, we found a significant interaction effect of AD–rs2241894 genotype on plasma ADH1C level (*p* = 0.04) (Figure 2B). This interaction effect was attributable to the association between AD and plasma ADH1C level (β estimate = 366, 95% CI 92.7∼639.4, *p* = 0.009).

## Discussion

Our study demonstrated a suggestive association between AD and *ADH1C* rs2241894 genotypes in a recessive fashion. To the best of our knowledge, this is the first study to propose *ADH1C* rs2241894 genotypes as a protective factor of AD in the Taiwanese female population. Although there was a correlation between AD and *ADH1C* rs2241894 in the pilot study, the result in the extension study was not significant in both sexes, which may indicate the possibility of other confounding factors, such as age and lifestyle. This study did not find associations between AD and *ADH1B* (rs1229984), *ALDH1B1* (rs2073478), and *ALDH2* (rs886205, rs4767944, rs4648328, and rs671), indicating that *ADH1B*, *ALDH1B1*, and *ALDH2* played no role in the relationship between alcohol and AD.

Alcohol elimination was catalyzed by ADH and ALDH (Zakhari, 2006). Class I ADH, consisting of several homo- and heterodimers of alpha, beta, and gamma subunits, exhibits high activity for ethanol oxidation to acetaldehyde, thus playing a major role in ethanol catabolism (Cederbaum, 2012). *ADH1C* encodes the gamma subunit of class I ADH. *ADH1C* cytoplasmic expression was mainly observed in glandular cells of the gastrointestinal tract, including the liver, duodenum, and stomach. *ADH1B* and *ADH1C* have polymorphisms that produce isoenzymes with distinct kinetic properties. Previous studies showed that the genetic variations in ADH genes were related to alcohol consumption (Edenberg and Foroud, 2013); however, this was more evident for the ADH2 gene, whereas the *ADH1C* polymorphism, as in our study, showed a small influence on the risk of alcoholism. In a previous SNP study, *ADH1C* was associated with alcohol elimination rate, whereas rs2241894 was not associated with the *in vivo* kinetic model of alcohol metabolism (Birley et al., 2009).

Alcohol metabolism via ADH produces acetaldehyde and oxygen radicals, which are highly reactive molecules (Zakhari, 2006). Neurons are extremely sensitive to attacks by destructive free radicals. In the brains of AD patients, free radical leads to DNA damage, protein oxidation, lipid peroxidation, and advanced glycosylation, which further aggravates AD pathology including neurofibrillary tangles and senile plaques (Tonnies and Trushina, 2017).

The genetic distribution of *ADH1C* rs2241894 showed strong ethnic heterogeneity, in which the T allele was the minor allele accounting for 28.5% in our study, 23.6% in East Asians, and 40% in South Asians, while it was a major allele in Americans (83.0%), Europeans (76.5%), and the global populations (52.8%) (Huang et al., 2020). *ADH1C* rs2241894 (A > G, synonymous variant Thr151, exon 5) is a synonymous variant. Moreover, we did not find functional SNPs that have LD with rs2241894 on SNPsnap^2^. To the best of our knowledge, there is no report showing an association between AD and rs2241894 or nearby SNPs.

Differences in drinking habit and alcohol metabolism exist between sex. In the United States, epidemiological evidence suggests that nearly 20% of adult males suffer from alcohol abuse or alcoholism-related complications. On the other hand, only approximately 5–6% of adult females are alcoholic or abuse alcohol on a regular basis (Mumenthaler et al., 1999). In Asia, men are prone to alcohol drinking in contrast to women (Millwood et al., 2019), in which we have demonstrated that the rate of alcohol consumption was 0% in women versus 26% in men (Chen et al., 2006, 2009). In addition, the toxic effect of alcohol can be influenced by genes; for example, men carrying APOE ε2ε3 have a greater tendency to suffer from strokes than those with ε3ε3 when they have alcohol exposure (Chen et al., 2009). In alcoholic pharmacokinetics, women have increased bioavailability and a faster clearance rate (Mumenthaler et al., 1999). Another example of alcohol–gene interaction is the class III ADH (glutathione-dependent ADH). Women develop higher blood alcohol levels than men in spite of an equal alcohol intake due to a smaller gastric metabolism in women due to the lesser activity of class III ADH in females (Baraona et al., 2001). Therefore, sex differences in the effects of alcohol metabolism on AD should be tested to illuminate the genetic roles of AD in personalized management (Sultatos et al., 2004).

The association between AD and *ADH1C* rs2241894 was significant in the pilot study but not in the extension study. The conflicting findings may be due to the difference between the two control groups. The controls were older and had more comorbidities in the pilot study than the extension study, but similar to the AD patient group. The subjects from the Taiwan Biobank were younger and healthier, and may have better lifestyles, such as social activities, exercise, and diet. In addition, the number of patients was relatively small compared with the control number in the extension study. The positive result may be caused by a statistical effect.

## Limitation

This study is the first to discuss the association between *ADH1C* rs2241894 and AD under sex disparities. However, there are some limitations to our study. First, alcohol intake was much lower in Asian females than in males; therefore, the sample size was small, especially for those with alcohol use. Second, the frequencies of alcohol-metabolizing genes differ among ethnicities (Huang et al., 2020). Besides, the sizes of the examined samples are small and have limited power to detect genetic association of minor/modest effect with AD. The discrepancy in the results of pilot and extension study may be caused by age difference between two groups. The result should be interpretated with caution and further studies with age-matched and larger sample size were indicated for further confirmation of the results herein.

## Conclusion

This study revealed a suggestive association between the genetic variant of *ADH1C* rs2241894 and female AD in Taiwanese population. Carrying the *ADH1C* rs2241894 TT genotype may be a protective factor for elderly female Taiwanese individuals.

## Data Availability Statement

The datasets presented in this study can be found in online repositories. The names of the repository/repositories and accession number(s) can be found in the article/Supplementary Material.

## Ethics Statement

The studies involving human participants were reviewed and approved by the Institutional Review Board/Ethics Committee (IRB/EC) protocol was approved by the Medical Ethics Committee of Chang Gung Memorial Hospital, and the ethical approval code was IRB 201700444B0C602. The ethical approval for the study was granted by the IRB of the Taiwan Biobank before the study was conducted (approval number: 201506095RINC). The patients/participants provided their written informed consent to participate in this study.

## Author Contributions

Y-CC: conceptualization, methodology, investigation, resources, data curation, review and editing, project administration, and funding acquisition. Y-YW: methodology, formal analysis, and writing – original draft. Y-SL: resources, bioinformatics, data acquisition, and formal analysis. Y-LL: resources and data acquisition. W-CH, W-MH, and Y-HH: patient enrollment. S-JT: data acquisition and review and editing. P-HK: data acquisition and review and editing. All authors read and approved the final manuscript.

## Conflict of Interest

The authors declare that the research was conducted in the absence of any commercial or financial relationships that could be construed as a potential conflict of interest.

## Footnotes

1 https://taiwanview.twbiobank.org.tw/index

2 https://data.broadinstitute.org/mpg/snpsnap/
