# Supplementary material for: Association Study of Alcohol Dehydrogenase and Aldehyde Dehydrogenase Polymorphism With Alzheimer Disease in the Taiwanese Population
Source: Front Neurosci. 2021 Jan 22;15:625885. doi: 10.3389/fnins.2021.625885 (PMC7862325; doi:10.3389/fnins.2021.625885)
Supplement: Supplementary file 1 [file Table_1.doc]

Supplement 1 Association of candidate SNPs with AD in pilot case-control study

Model 1: adjust age, education, HTN, DM

Model 2: adjust age, education, HTN, DM and alcohol

|  | Male | | | | | | Female | | | | | |
| --- | --- | --- | --- | --- | --- | --- | --- | --- | --- | --- | --- | --- |
|  | dominant | | additive | | recessive | | dominant | | additive | | recessive | |
| SNP | model 1 | model 2 | model 1 | model 2 | model 1 | model 2 | model 1 | model 2 | model 1 | model 2 | model 1 | model 2 |
| rs1229984 | 0.50 | 0.50 | 0.23 | 0.23 | 0.12 | 0.12 | 0.04 | 0.04 | 0.07 | 0.07 | 0.99 | 0.99 |
| rs2241894 | 0.49 | 0.49 | 0.45 | 0.45 | 0.61 | 0.61 | 0.26 | 0.26 | 0.04 | 0.04 | 0.01 | 0.01 |
| rs2073478 | 0.66 | 0.66 | 0.52 | 0.52 | 0.48 | 0.48 | 0.25 | 0.25 | 0.83 | 0.83 | 0.15 | 0.15 |
| rs886205 | 0.79 | 0.79 | 0.77 | 0.77 | 0.84 | 0.84 | 0.19 | 0.19 | 0.08 | 0.08 | NA | NA |
| rs4767944 | 0.21 | 0.21 | 0.43 | 0.43 | 0.64 | 0.64 | 0.66 | 0.66 | 0.39 | 0.39 | 0.24 | 0.24 |
| rs4648328 | 0.34 | 0.34 | 0.45 | 0.45 | 0.88 | 0.88 | 0.71 | 0.71 | 0.92 | 0.92 | 0.53 | 0.53 |
| rs671 | 0.99 | 0.99 | 0.69 | 0.69 | 0.41 | 0.41 | 0.39 | 0.39 | 0.55 | 0.55 | 0.91 | 0.91 |

*P*-value with Bonferroni correction for significance was 0.007 in pilot case-control study

Supplement 2 Association of candidate SNPs with AD in extension study

Model 1: adjust age, education, HTN, DM

Model 2: adjust age, education, HTN, DM and alcohol

|  | Male | | | | | | Female | | | | | |
| --- | --- | --- | --- | --- | --- | --- | --- | --- | --- | --- | --- | --- |
|  | dominant | | additive | | recessive | | dominant | | additive | | recessive | |
| SNP | model 1 | model 2 | model 1 | model 2 | model 1 | model 2 | model 1 | model 2 | model 1 | model 2 | model 1 | model 2 |
| rs1229984 | 0.55 | 0.46 | 0.57 | 0.41 | 0.06 | 0.58 | 0.79 | 0.78 | 1.00 | 1.00 | 0.59 | 0.59 |
| rs2241894 | 0.23 | 0.24 | 0.08 | 0.09 | 0.06 | 0.08 | 0.39 | 0.39 | 0.29 | 0.29 | 0.72 | 0.72 |
| rs2073478 | 0.13 | 0.12 | 0.35 | 0.34 | 0.74 | 0.72 | 0.12 | 0.12 | 0.40 | 0.40 | 0.77 | 0.77 |
| rs886205 | 0.40 | 0.48 | 0.15 | 0.19 | NA | NA | 0.51 | 0.51 | 0.46 | 0.46 | 0.60 | 0.60 |
| rs671 | 0.91 | 0.57 | 0.80 | 0.39 | 0.45 | 0.29 | 0.65 | 0.65 | 0.56 | 0.56 | 0.59 | 0.59 |

*P*-value with Bonferroni correction for significance was 0.01 in extension study
